# The Mu.Ta.Lig. Chemotheca: A Community-Populated Molecular Database for Multi-Target Ligands Identification and Compound-Repurposing

**DOI:** 10.3389/fchem.2018.00130

**Published:** 2018-04-19

**Authors:** Francesco Ortuso, Donatella Bagetta, Annalisa Maruca, Carmine Talarico, Maria L. Bolognesi, Norbert Haider, Fernanda Borges, Sharon Bryant, Thierry Langer, Hanoch Senderowitz, Stefano Alcaro

**Affiliations:** ^1^Department of Health Sciences, Magna Græcia University of Catanzaro, Catanzaro, Italy; ^2^Department of Pharmacy and Biotechnology, Università di Bologna, Bologna, Italy; ^3^Department of Pharmaceutical Chemistry, Faculty of Life Sciences, University of Vienna, Vienna, Austria; ^4^Department of Chemistry and Biochemistry, Faculty of Sciences, CIQUP, Universidade do Porto, Porto, Portugal; ^5^Inte:Ligand GmbH, Vienna, Austria; ^6^Department of Chemistry, Bar-Ilan University, Ramat Gan, Israel

**Keywords:** molecular database, multi-target drugs, drug repurposing, LAMP server, openbabel, Pybel, molecular descriptors, scientific collaboration

## Abstract

For every lead compound developed in medicinal chemistry research, numerous other inactive or less active candidates are synthetized/isolated and tested. The majority of these compounds will not be selected for further development due to a sub-optimal pharmacological profile. However, some poorly active or even inactive compounds could live a second life if tested against other targets. Thus, new therapeutic opportunities could emerge and synergistic activities could be identified and exploited for existing compounds by sharing information between researchers who are working on different targets. The Mu.Ta.Lig (Multi-Target Ligand) Chemotheca database aims to offer such opportunities by facilitating information exchange among researchers worldwide. After a preliminary registration, users can (a) virtually upload structures and activity data for their compounds with corresponding, and eventually known activity data, and (b) search for other available compounds uploaded by the users community. Each piece of information about given compounds is owned by the user who initially uploaded it and multiple ownership is possible (this occurs if different users uploaded the same compounds or information pertaining to the same compounds). A web-based graphical user interface has been developed to assist compound uploading, compounds searching and data retrieval. Physico-chemical and ADME properties as well as substructure-based PAINS evaluations are computed on the fly for each uploaded compound. Samples of compounds that match a set of search criteria and additional data on these compounds could be requested directly from their owners with no mediation by the Mu.Ta.Lig Chemotheca team. Guest access provides a simplified search interface to retrieve only basic information such as compound IDs and related 2D or 3D chemical structures. Moreover, some compounds can be hidden to Guest users according to an owner's decision. In contrast, registered users have full access to all of the Chemotheca data including the permission to upload new compounds and/or update experimental/theoretical data (e.g., activities against new targets tested) related to already stored compounds. In order to facilitate scientific collaborations, all available data are connected to the corresponding owner's email address (available for registered users only). The Chemotheca web site is accessible at http://chemotheca.unicz.it.

## Introduction

The Chemotheca database was developed within the framework of the COST ACTION CA15135, “MuTaLig.” It is focused on the identification of multi-target ligands and on the possibility for repurposing such bio-active compounds. These goals could be reached by stimulating new scientific collaborations among research groups involved, in various ways, in the study of drugs, and by merging their results. In fact, large numbers of molecules with a potential pharmaceutical relevance are developed in universities and in pharmaceutical industry each year. Most of these compounds will never reach the market due to some failure in pharmacology profile. Nevertheless, these chemical entities and their pharmacological profiles can be considered as a source of information evaluable against other targets. With this in mind, an information exchange platform, designed to allow for a direct connection among its users, has been developed. In contrast with already available services, such as ZINC (Irwin and Shoichet, 2005; Irwin et al., 2012), ChEMBL (Bento et al., 2014), PubChem (Kim et al., 2016), ChemSpider (www.chemspider.com), DrugBank (Wishart et al., 2006, 2018), WOMBAT (Good and Oprea, 2008), DUD (Huang et al., 2006), CSD (Groom et al., 2016), and others, which typically provide only compound download options, the Chemotheca database permits registered users to directly upload their own data. Such information immediately becomes available on the web site and contains the corresponding owner's contact details. Chemotheca software, automatically and user transparently, checks if the uploading compound structure has been designed according to chemistry rules and if it is consistent with mandatory fields required data. Additional information includable with uploaded compounds can be both experimental and theoretical and contain attributes such as molecular structures, activities, ADME/t properties, physico-chemical descriptors and references. For each new uploaded compound, 90 molecular descriptors are computed, on the fly. To safeguard intellectual property, and to facilitate collaboration among participants, the (user)name and the email address of the user who has uploaded the data are shown. To prevent abuse (such as spam), these details are disclosed to registered users only. A very detailed query form has been developed for searching deposited records. Matching query results can be exported as standard SMILES, SDF, MOL2 and HTML file formats.

## Materials and methods

The Chemotheca was developed with open-source programming environments, its core being a web-accessible molecular database. It is hosted at the Magna Græcia University of Catanzaro (Italy) by the Medicinal Chemistry laboratory. An Intel Xeon 64 bit dual processor cluster facility, running under the Linux CentOS 7 (www.centos.org) operating system, offers the service. Apache software (www.apache.org) serves the web site, which complies with the W3C DTD HTML 4 standard (www.w3.org). Static and dynamic web pages have been written using the PHP programming language (www.php.net). The advanced search form and the compound upload user interfaces include the JSME molecular editor (Bienfait and Ertl, 2013). All Chemotheca data are stored in a MySQL database (www.mysql.com). An *ad hoc* developed Python (www.python.org) code (CDC) computes molecular descriptors for each uploaded compound, by using the OpenBabel library (O'Boyle et al., 2011) and its wrapper Pybel (O'Boyle et al., 2008).

The molecule upload process uses the isomeric SMILES code produced by the JSME applet. The CDC converts SMILES into a fingerprint. Fingerprint pattern matching prevents duplicate structures. If the compound is already available, another (new) owner will be added only. In case of a new structure entry, SMILES is converted into 2D and 3D SDF and MOL2 file formats. The 3D structure atom coordinates are optimized using the MMFF94 force field *in vacuo*. The energy minimization protocol consists of 150 steps of Steepest Descent algorithm followed by variable steps of Conjugate Gradients (50 for each rotatable bond). A convergence criterion of 0.05 kcal/mol·Å^−1^ is used. Molecular descriptors, such as Lipinski rule compliance, CNS bioavailability, PAINS matching, LogBB, LogP, total polar surface area (TPSA), molecular weight, are computed using OpenBabel and Pybel built-in functions. The Central Nervous System (CNS) bioavailability and the LogBB descriptors are estimated according to Vilar et al. (2010). The Pan Assay INterference compoundS (PAINS) detection is based on the Pybel Smarts comparison between the new compound and the substructures reported by Baell and Holloway (2010). Users should carefully evaluate the application of any PAINS filtering because of the controversial opinions in the literature (Aldrich et al., 2017; Capuzzi et al., 2017). Search procedures are based on MySQL queries that are built and executed by the PHP code of the “Simple search” or “Advanced search” forms.

## Discussion

The Chemotheca has been developed with the aims of (a) identifying multi-target agents and repurposing known active compounds and (b) stimulating new scientific collaborations between researchers while saving intellectual property of all involved researchers. The success of this approach will depend not only on its unique concept and the quality of its infrastructure, but mainly on its visibility to the scientific community and on its attractiveness for researchers to join.

### Registration

**Figure 1:**
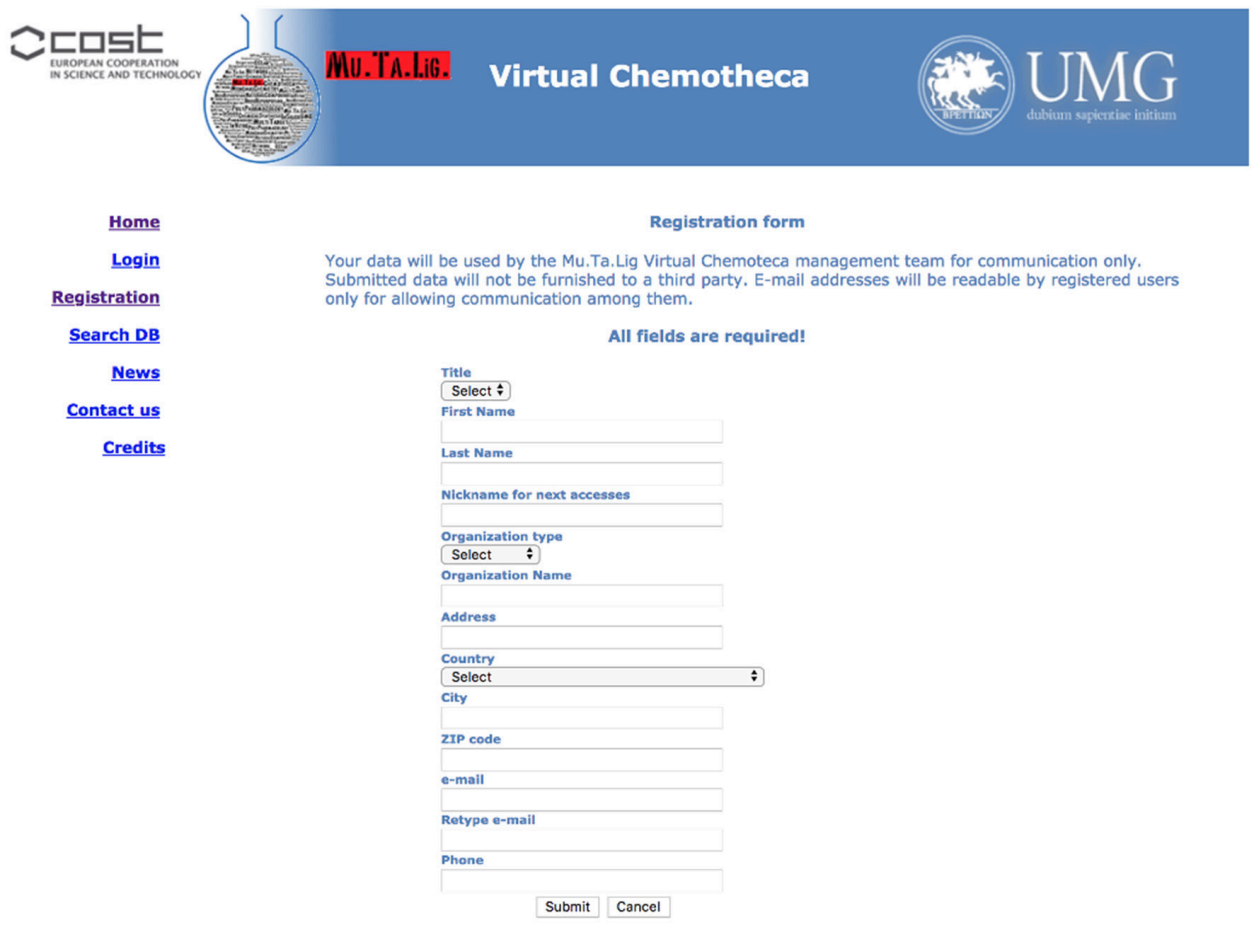
Registration form.

The users' registration is crucial for the Chemotheca purposes and it is strongly encouraged. Guest access is allowed but it is very limited in terms of offered functionalities: (a) the upload of new information is not possible, (b) the advanced search form is not available, and (c) the search results consist of a list of molecular structures only. Basically, guest access should be considered for the very first visit to the web site. The registration procedure has been designed to be as easy as possible: a predefined form has to be filled with minimal personal information and, after its submission, an automatic email message will summarize the registration data and will communicate the password for the first authenticated access. After the login, the “Your Profile” menu allows the update of the personal information and the management of frequently used search queries, if they have been previously saved (Figure 1).

### Search facilities

**Figure 2:**
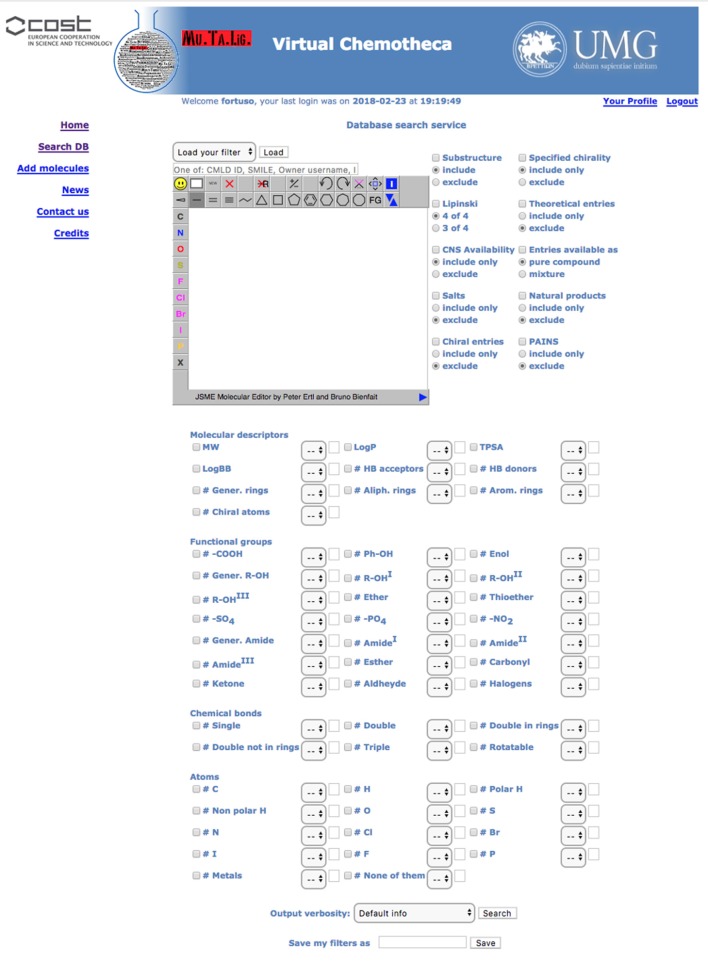
Advanced search form.

All users can search for stored compounds by using the simple search query form. The advanced search interface is available for registered users only. In simple search, SMILES notation, owner username, InChI, InChIKey or, if known, the compound identity code (ID) can be, singularly, applied as query items. The advanced search interface offers many more options linked together by the Boolean operator AND. In addition to simple search criteria, the advanced query form can take into account (a) chemical substructure, (b) owner furnished compound's features such as chirality, origin (natural, organic synthesis or theoretical), purity, (c) Chemotheca computed properties, and (d) functional groups, chemical bonds and atom type. Before submitting the search, the user can save the query in the “Your Profile” menu to be reloaded for further investigation. The results detail level can be customized (Figure 2).

Results are displayed, as a pageable table reporting the 2D chemical structure of matching compounds, their owner(s) and properties according to the detail level previously selected. By default, 10 rows per page will be shown, but the user can modify this parameter. By clicking on structures, these will be magnified and all related properties will be displayed in a new popup window. “Edit query” and “Re-filter hits” options are available to improve search efficacy and focus the results. The first option from the results table returns to the search form where the previous query appears. This query could be modified and used for searching again the entire database. The “Re-filter hits” appears similar but the modified query will only be applied to the previous results.

### New compound upload

**Figure 3:**
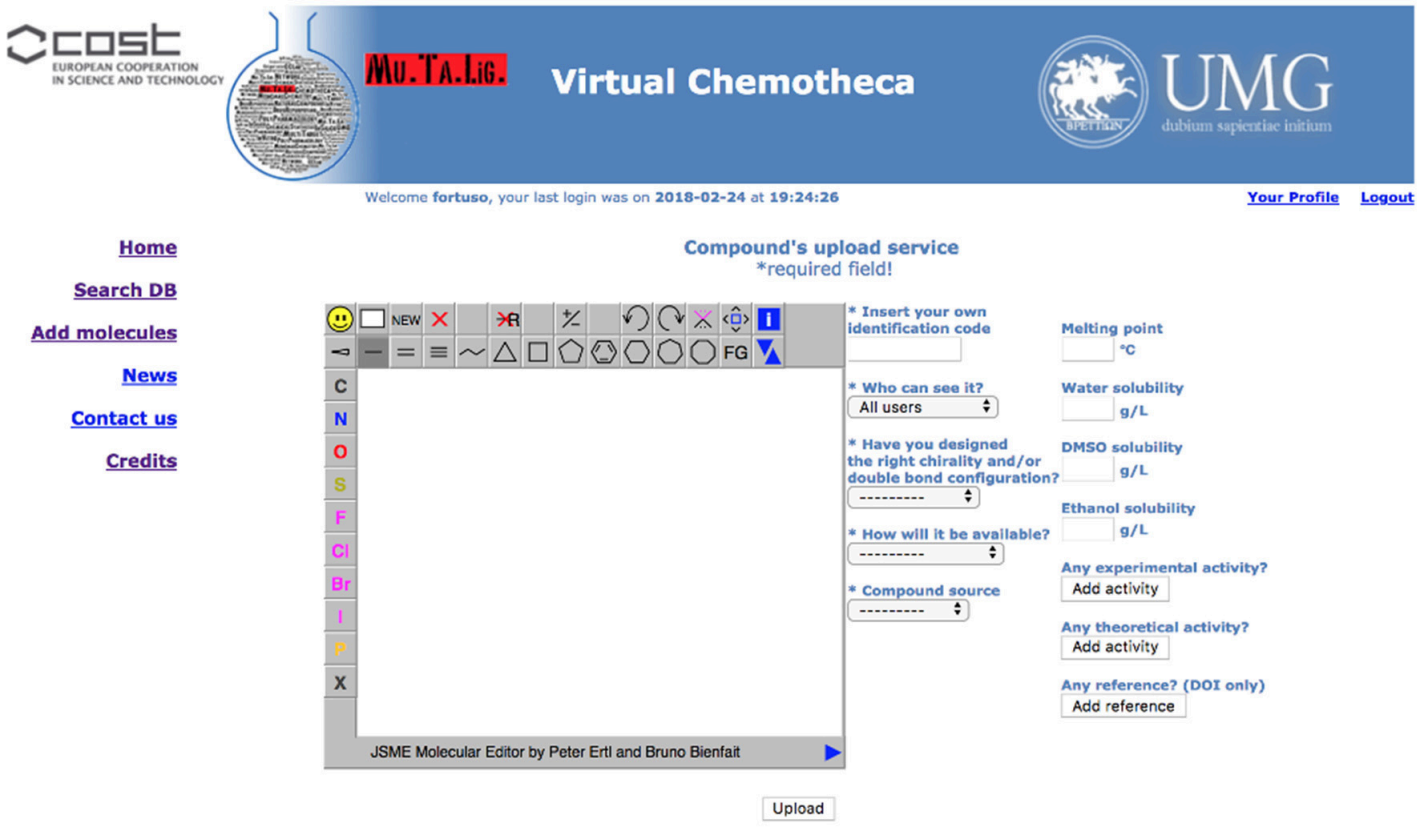
New data upload service.

Registered users can add their own compounds and/or their own data to existing compounds. A specific form has been developed and is hidden to guest users (Figure 3).

Structures for uploaded compounds must be drawn with the embedded JSME editor. The user has to fill in four mandatory fields: (a) a unique molecule identification code, which will help if other researchers contact the owner, (b) whether the structure has been properly designated with respect to chirality and double bonds configuration, (c) how the molecule will be delivered, i.e., pure compound, mixture of isomers, or theoretical (of course, a theoretical entry cannot be delivered and should be considered as an inspiration source for researchers involved in chemical synthesis), and (d) the origin of the molecule, i.e., natural or synthetic. Fields (b) and (c) will be taken into account together for verifying the data coherence and to prevent low quality information: Chemotheca allows the upload of theoretical or pure compounds if their chemical structures explicitly report chirality and double bond configuration. This is not mandatory for compounds delivered as a mixture. By using the corresponding menu, both experimental and theoretical information, and references can be added. These data can be included also for already available structures, owned by different users. In this case, the new properties will be linked to the molecular structure and a new ownership, related to the corresponding information, will be added.

### Further development

The Chemotheca has reached a stable state of development. At present, the web interface allows for a single-compound upload only. This is a limitation when several/multiple structures need be added. At the moment, it is possible to overcome such a limitation by filling in a spreadsheet template furnished by the Chemotheca's developers. In the near future, a multiple compounds upload facility will be implemented. Another feature under development is the possibility, for the corresponding owner, to edit the information included in the database. For now the data editing, excluding the personal information, is possible only by contacting the developers. Finally, a new advanced search form is under development: it will allow the database search by using activities as query criteria. The Chemotheca's developer team is open to suggestions and offers to contribute.

## Conclusion

According to the mission of COST ACTION CA15135, a computational platform has been developed for stimulating scientific collaboration among research groups worldwide, focused on multi-target ligands identification and compound-repurposing. Already about one hundred researchers are registered and are frequently using the system to search and upload compounds and related information.

## Author contributions

FO: Coordinator and Software developer; DB: Software debugging and database population; AM: Software debugging and database population; CT: Software developer; MB: Software features design; NH: Software features design; FB: Software feature design and database population; SB: Software debugging; TL: Software design and debugging; HS: Software design and debugging; SA: Software design and debugging.

### Conflict of interest statement

SB is affiliated with Inte:Ligand GmbH and declares no competing interests. The other authors declare that the research was conducted in the absence of any commercial or financial relationships that could be construed as a potential conflict of interest.
